# *Clerodendrum trichotomum* Thunberg—An Ornamental Shrub with Medical Properties

**DOI:** 10.3390/molecules29143272

**Published:** 2024-07-10

**Authors:** Jan Gomulski, Izabela Grzegorczyk-Karolak

**Affiliations:** Department of Biology and Pharmaceutical Botany, Medical University of Lodz, 90-151 Lodz, Poland; jan.gomulski@umed.lodz.pl

**Keywords:** acteoside, anti-inflammatory activities, Chou-Wu-Tong, diterpenoids, hypotensive effect, Lamiaceae, phytoconstituents

## Abstract

Harlequin glory bower (*Clerodendrum trichotomum*) is a shrub or small tree belonging to the Lamiaceae family, native to Japan, Korea, and eastern China. It has esthetic value and in Europe, it is cultivated as an ornamental plant. Its sweet-smelling flowers have a white or pink crown. The calyx turns from green to pink–purple over time, providing an especially decorative touch around surrounding the ripe deep-blue fruits that persist until winter. In the areas of its natural occurrence, the leaves and young shoots of *C. trichotomum*, and sometimes the roots, flowers and fruits, are used in folk medicine due to its anti-inflammatory, analgesic, anticancer, sedative, and hypotensive effects. Products based on Harlequin glory are also used in the treatment of rheumatoid arthritis, joint pain, skin inflammation, or asthma. Preliminary research on the composition of raw material suggests that its health-promoting effect is associated with the presence of numerous secondary metabolites, including phenylpropanoids, flavonoids, lignans, terpenoids, steroids, alkaloids, and anthraquinones. This work reviews the current state of knowledge about the phytochemistry and in vitro and in vivo pharmacological properties of the extracts and main active components isolated from *C. trichotomum*. It also indicates that before it can be used in modern medicine, further research is necessary regarding the safety and efficacy of the raw material, its mechanisms of action, and dosage.

## 1. Introduction

The use of conventional drugs is often supplemented by the use of medicinal plants as therapeutic agents, with herbal remedies being more available and less expensive. According to the World Health Organization (WHO), herbal medicines meet the healthcare needs of approximately 80% of the world’s population, especially in developing countries [1]. Although medicinal plants have been used in treatments for millennia, scientific research on their beneficial effects has gained popularity only in the last few decades. Such research is particularly poor concerning species outside of traditional European medicine.

One particularly diverse taxon that has been utilized in traditional and folk medicine for centuries in India, China, Japan, Thailand, and some areas of Africa is the genus *Clerodendrum*. Dozens of species from this genus have been used as anti-inflammatory, antidiabetic, antihypertensive, antimalarial, antiviral, hypolipidemic, antioxidant, and anticancer agents [2,3,4,5,6,7]. However, despite the seemingly promising potential, only a few of these species have been studied so far with regard to their chemical composition and pharmacological properties.

The genus *Clerodendrum* L. initially belonging to the Verbenaceae family, according to the APG system (Angiosperm Phylogeny Group), has been assigned to the Lamiaceae [8,9]. It was first described by Linnaeus in 1753, based on the species *Clerodendrum infortunatum* found in India. Although the name was changed to Clerodendron (from the Greek words: klero–chance and dendron–tree) ten years later, the Latin name was reintroduced in 1942 by Moldenke and is currently the most commonly used [10].

*Clerodendrum* is a genus comprising about 580 species, including small trees, shrubs, and herbaceous plants. They are primarily found in the tropical and subtropical zones of Asia, Australia, America, and Africa [2,10]. Several species can be found in the temperate climate zone, but they are primarily cultivated as ornamental plants. One of the species, sometimes found in gardens around Europe due to its relative frost resistance, is the species from Japan and China: *C. trichotomum*.

The aim of this paper is to present the current state of knowledge about *C. trichotomum*, with particular emphasis on its botanical characteristics, chemical composition, and traditional use, as well as the biological activity of its raw materials and bioactive compounds. This review also aims to generate interest in this intriguing and beautiful species, which remains relatively unknown in Europe. Promising results from studies on its composition and activity encourage further efforts to deepen our understanding of this species. This is particularly important because modern requirements for medicinal raw materials necessitate the use of only detailed, tested, and standardized products, whereas there are no established official therapeutic indications, administration forms, dosages, or safety profiles for *C. trichotomum* and its bioactive compounds.

## 2. Methodology of Paper Selection

The papers were selected from the following electronic databases: Google Scholar, scientific databases (PubMed, Scopus, and Web of Science), and various publishers as well as Flora of China using different relevant keywords. The following search phrases were used: “*Clerodendrum trichotomum*”, “morphology of *Clerodendrum trichotomum*”, “*Clerodendrum* extracts”, “phytochemisty of *Clerodendrum*”, “properties of *Clerodendrum*”, “biological activities of *Clerodendrum*”, “phytoconstituents of *Clerodendrum*”, “taxonomy of *Clerodendrum*”, toxicology of *Clerodendrum*”, ethnopharmacology of *Clerodendrum*”, and “medicinal uses of *Clerodendrum*”. Searches were conducted without imposing any language restrictions, but papers published in languages other than English without an available English abstract were rejected.

Ultimately, 39 published reviews and experimental studies from the period 1970–2022 were selected, of which 11 concerned the isolation of bioactive compounds from *C. trichotomum*, 11 biological activity of *C. trichotomum* extracts and its secondary metabolites, 13 were a combination of phytochemistry and biological activity of the species, one was related to the morphology of the species, and three were reviews of several plants from the *Clerodendrum* genus, including this species. Most biological studies were based on simple in vitro tests. Studies conducted on animals were few and only fragmentary, and none were clinical studies. 

Among the activity studies, only papers describing products (infusions, extracts, and the fractions obtained from them) and compounds isolated directly from *C. trichotomum* were included. Studies involving synthetic compounds were excluded, as well as studies reporting the compounds isolated from other plant species known to be present in *C. trichotomum*. This applies especially to acteoside, a compound with such numerous, widely-documented biological activities that it is even the subject of separate reviews [11,12,13].

## 3. Distribution and Morphology of *C. trichotomum*

*Clerodendrum trichotomum* (Harlequin glory bower, Chance tree, Japanischer Losbaum, Chou-Wu-Tong) occurs naturally in lowland and mountainous areas in Japan, Korea, and eastern China [2], where it grows as a shrub or small tree, reaching heights of 1.5–10 m. In Europe, it rarely reaches a tree-like form, and when planted as an ornamental plant in gardens, it usually does not exceed 2 m in height.

The stems and leaves of *C. trichotomum* have soft hairs and emit an unpleasant odor when crushed. The leaves are opposite, dark green, up to 20 cm long, and variable in shape: ovate-elliptic, triangular-ovate, or ovate, with a broadly cuneate, truncate, or, rarely, heart-shaped base and a sharply pointed apex. The length of the leaf petioles ranges from 2 to 8 cm [14]. The plant blooms from August to October. Inclined dichotomous inflorescences, 8–20 cm long, appear at the ends of branches. Each individual flower has a diameter of about 2–3 cm, emits a sweet fragrance, and its corolla is white or pinkish. The calyx is greenish but gradually turns pink–purple. It is deeply lobed with five distinct lobes, triangular, lanceolate, or ovate in shape, pointed at the ends. The fruits are round and about 6–8 mm in diameter. Ripe ones are deep blue in color [14]. Surrounded by a pink, persistent, enlarged calyx, they look particularly decorative and persist until winter. However, in colder climate zones, the plants may not produce any fruits at all.

**Figure 1:**
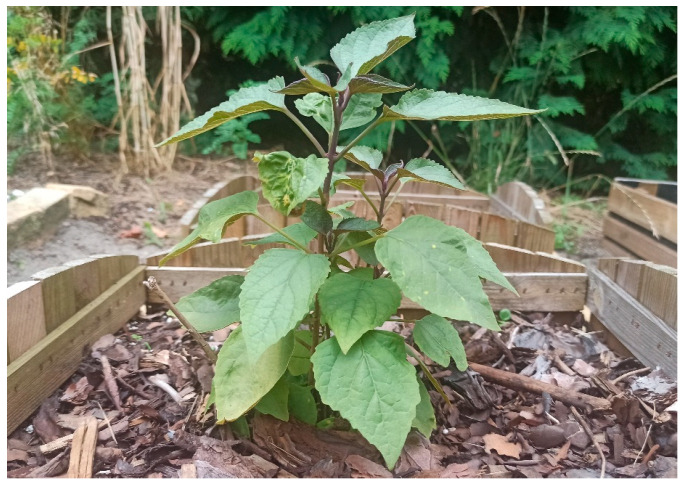
*Clerodendrum trichotomum*: a two-year-old plant transplanted in the spring in the current growing season from a pot to the ground.

A plant cultivated in greenhouse conditions and transplanted three months earlier at the beginning of the third growing season into the garden in Central Europa is given in Figure 1.

## 4. Applications in Traditional Folk Medicine

In China, and other regions where the plant grows wild, preparations from the leaves, stems, flowers, roots and fruits of *C. trichotomum* have been used for centuries in folk medicine [15]. Reports indicate that the leaves and stems demonstrate significant anti-inflammatory activity [3,4] and are applied in the treatment of inflammatory skin conditions. In Chinese medicine, the raw material is also recommended for eczema. It has anti-itching and mildly analgesic properties [4,15]. *C. trichotomum* has been recommended for the treatment of malaria and dysentery [8]. The leaves of the plants have anti-rheumatic effects [4,16]. Decoctions are used in the treatment of rheumatoid arthritis, joint pain, numbness, and paralysis. Moreover, the species was used as an anti-asthmatic agent [2]. In folk medicine, *C. trichotomum* is used for hypertension due to its calming and hypotensive properties [4,6]. It is also believed to have anti-diabetic properties [2]. Additionally, there are reports that the fruits may have anticancer potential [17].

## 5. Overview of Bioactive Compounds Identified in *C. trichotomum*

**Figure 2:**
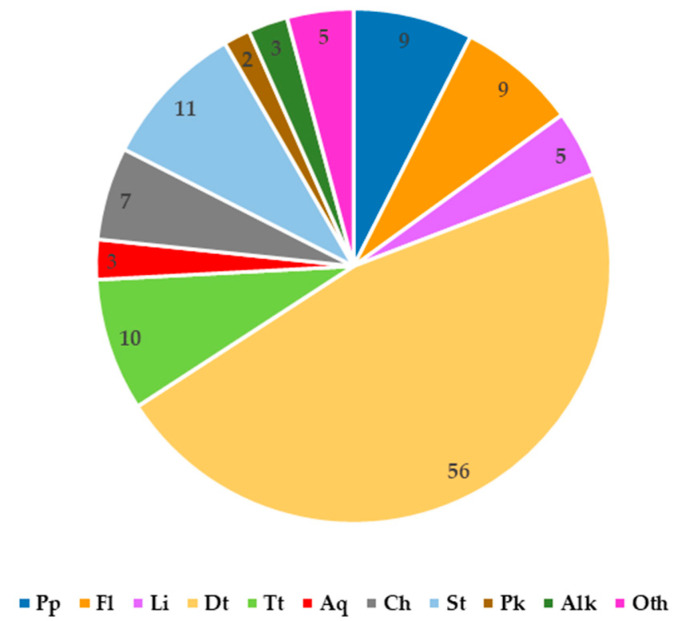
Number of secondary metabolites from various groups reported in *C. trichotomum*. Pp—phenylpropanoids; Fl—flavonoids; Li—lignans, Dt—diterpenoids, Tt—triterpenoids, Aq—anthraquinones; Ch—cyclohexylethanoids; St—steroids. Pk—polyketones; Alk—alkaloid; Oth—other compounds.

The first studies focusing on the chemical composition of *C. trichotomum* were carried out in the 1970s [18]. Since then, various phenolic compounds, including phenylpropanoids, flavonoids and lignans, terpenoids, steroids, alkaloids, anthraquinones, and essential oils have been detected in the plant [19] (Figure 2).

### 5.1. Phenolic Compounds

Phenolic compounds are widely represented in the genus *Clerodendrum* [2]. Their presence directly correlates with the biological activity of raw materials. Phenolic compounds may occur as free molecules or bound to sugar residues. The dominant group of phenolic compounds in *C. trichotomum* are phenylpropanoids.

#### 5.1.1. Phenylpropanoid Compounds

**Figure 3:**
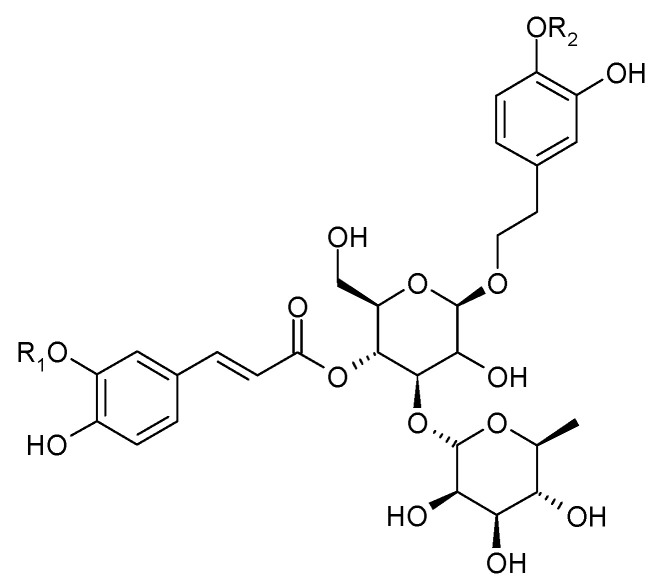
Chemical structures of phenylpropanoids present in *C. trichotomum*, part 1.

**Figure 4:**
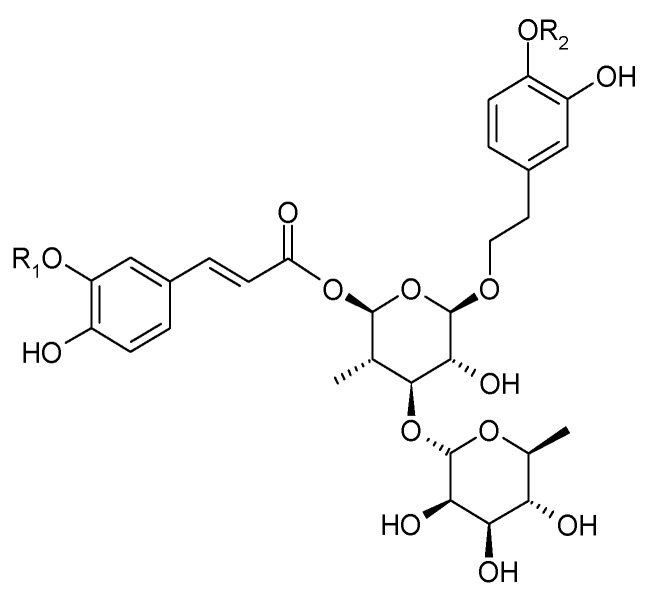
Chemical structures of phenylpropanoids present in *C. trichotomum*, part 2.

In 1983, Sukarai and Kato confirmed the presence of the most representative of the phenylpropanoids, acteoside (**1**) (verbascoside, kusaginin), in the leaves of the species [20]. In the early 21st century, apart from acteoside, others compounds from this group were isolated from the shoots of *C. trichotomum*: leucosceptoside (**2**), plantainoside C (**3**), jionoside D (**4**), martynoside (**5**), isomartynoside (**6**), and isoacteoside (**7**) (Figure 3 and Figure 4) [17,21].

(**1**) acteosideR_1_ = H;R_2_ = H(**2**) leucosceptosideR_1_ = CH_3_;R_2_ = H(**4**) jionoside DR_1_ = H;R_2_ = CH_3_(**5**) martynosideR_1_ = CH_3_;R_2_ = CH_3_

(**3**) plantainoside CR_1_ = CH_3_;R_2_ = H(**6**) isomartynosideR_1_ = CH_3_;R_2_ = CH_3_(**7**) isoacteosideR_1_ = H; R_2_ = H

**Figure 5:**
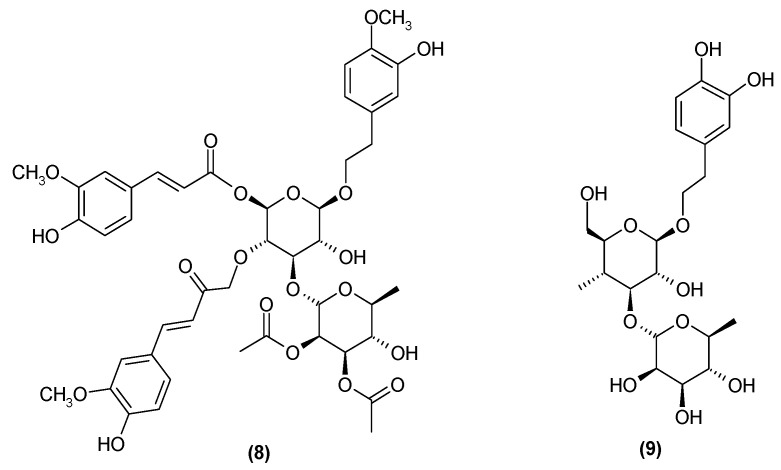
Chemical structures of phenylpropanoids present in *C. trichotomum*, part 3.

Subsequent studies have revealed the presence of two more phenylpropanoids: trichotomoside (**8**) [17] and decaffeoylacteoside (**9**) (Figure 5) [22].

(**8**) trichotomoside
(**9**) decaffeoylacteoside

#### 5.1.2. Flavonoids

**Figure 6:**
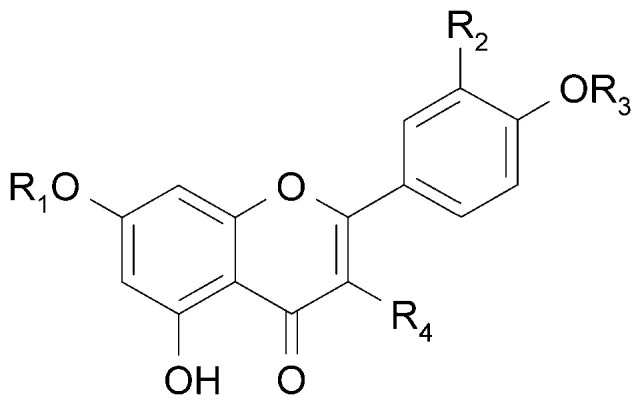
Chemical structures of flavonoids present in *C. trichotomum*, part 1.

**Figure 7:**
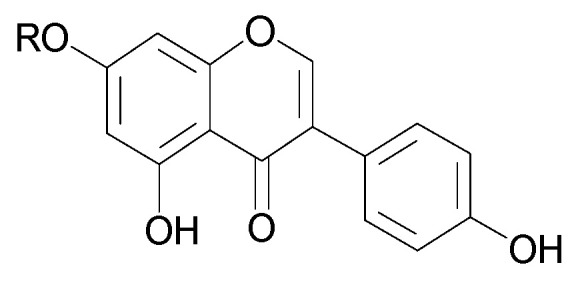
Chemical structures of flavonoids present in *C. trichotomum*, part 2.

Derivatives of 7-*O*-glucuronide of apigenin (**10a**) [23] and acacetin (**11**) have been isolated from the leaves of *C. trichotomum* [18]. In addition, seven other flavonoids have been identified in the flowers based on spectral data: apigenin (**12**) and its 7-*O*-glucoside (**10b**), genistein (**13**) and its 7-*O*-glucoside (**14**), chrysoeriol (**15**), kaempferol 3-*O*-glucoside (**16**), and isorhamnetin 3-*O*-glucoside (**17**) [24]. The above compounds are illustrated in Figure 6 and Figure 7.

(**10a**) apigenin-7-*O*-glucuronideR_1_ = glucuronic acid;R_2_ = H;R_3_ = H;R_4_ = H(**10b**) apigenin-7-*O*-glucosideR_1_ = glucose;R_2_ = H;R_3_ = H;R_4_ = H(**11**) acacetin-7-*O*-glucuronideR_1_ = glucuronic acid;R_2_ = H;R_3_ = CH_3_;R_4_ = H(**12**) apigeninR_1_ = H;R_2_ = H;R_3_ = H;R_4_ = H(**15**) chrysoeriolR_1_ = H;R_2_ = OCH_3_;R_3_ = H;R_4_ = H(**16**) kaempferol 3-*O*-glucosideR_1_ = H;R_2_ = H;R_3_ = H;R_4_ = glucose(**17**) isorhamnetin 3-*O*-glucosideR_1_ = H;R_2_ = OCH_3_;R_3_ = H;R_4_ = glucose

(**13**) genistein R = H(**14**) genistein 7-*O*-glucosideR = glucose

#### 5.1.3. Lignans

**Figure 8:**
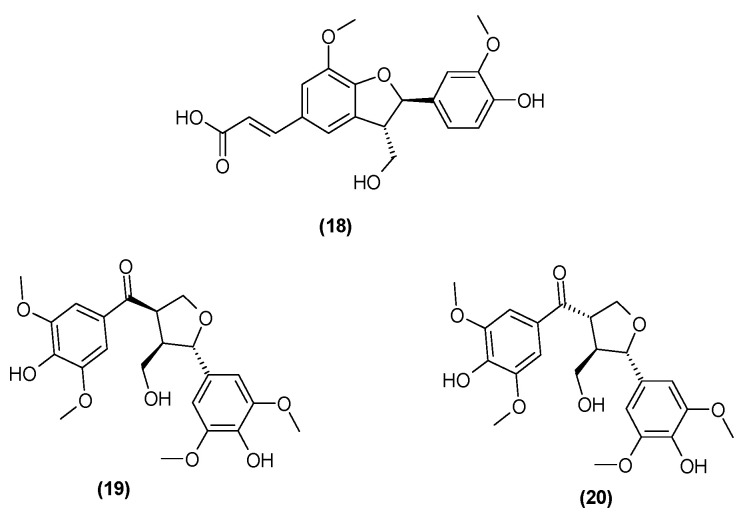
Chemical structures of lignans present in *C. trichotomum*, part 1.

**Figure 9:**
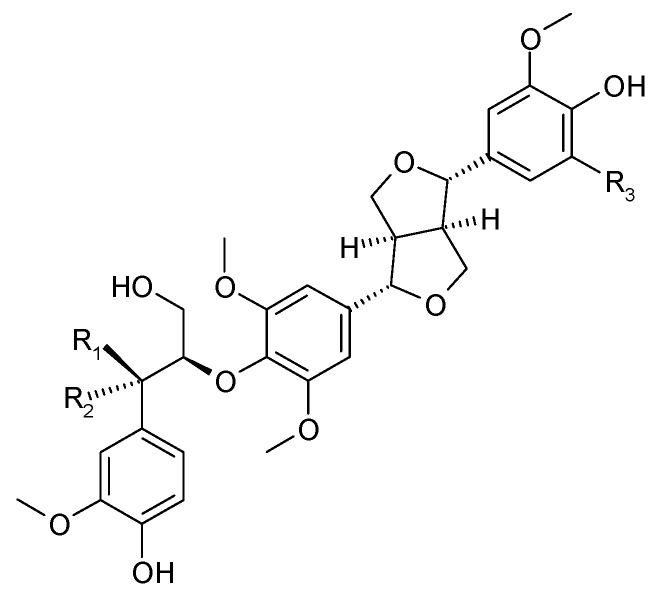
Chemical structures of lignans present in *C. trichotomum*, part 2.

Spicatolignan B (**18**) was isolated from the stems of *C. trichotomum* in 2014 [8]. Previously, this compound had only been identified in Lamiaceae in the herb of green mint [25]. In recent years, additional compounds from this group have been isolated from the leaves and branches: 5,5′-dimethoxy-7-oxolariciresinol (**19**) and (−)-(7′S,8S,8′R)-4,4′-dihydroxy-3,3′,5,5′-tetramethoxy-7′,9-epoxy-lignan-9′-ol-7-one (**20**) [26] (Figure 8). In 2021, the presence of ecdysanol D (**21**) and E (**22**) were also identified in the leaves of *C. trichotomum* (Figure 9) [26].

(**18**) spicatolignan B
(**19**) 5,5′-dimethoxy-7-oxolariciresinol
(**20**) (−)-(7′S,8S,8′R)-4,4′-dihydroxy-3,3′,5,5′-tetramethoxy-7′,9-epoxy-lignan-9′-ol-7-one

(**21**) ecdysanol DR_1_ = OH;R_2_ = H;R_3_ = H(**22**) ecdysanol ER_1_ = H;R_2_ = OH;R_3_ = OCH_3_

### 5.2. Terpenoids

#### 5.2.1. Diterpenoids

**Figure 10:**
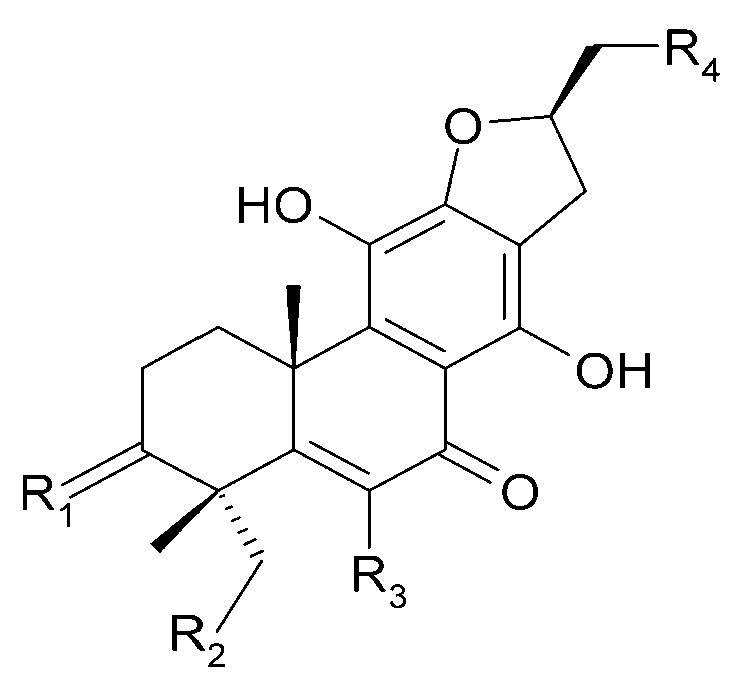
Chemical structures of diterpenoids present in *C. trichotomum*, part 1.

**Figure 11:**
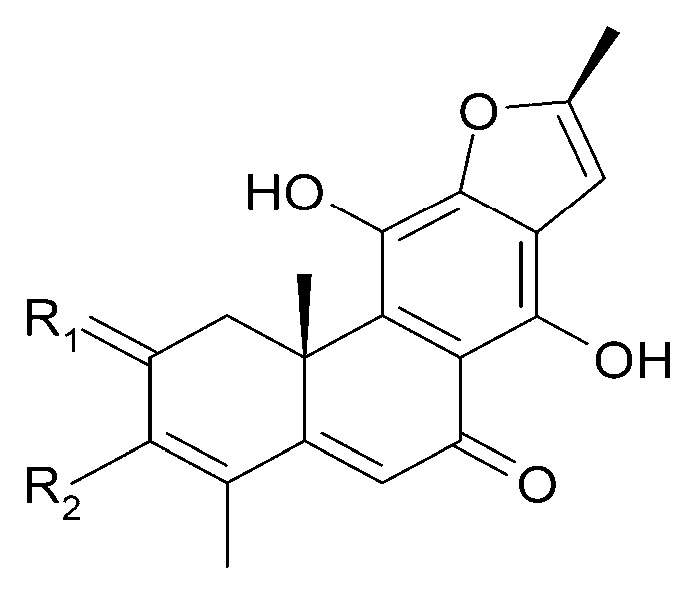
Chemical structures of diterpenoids present in *C. trichotomum*, part 2.

**Figure 12:**
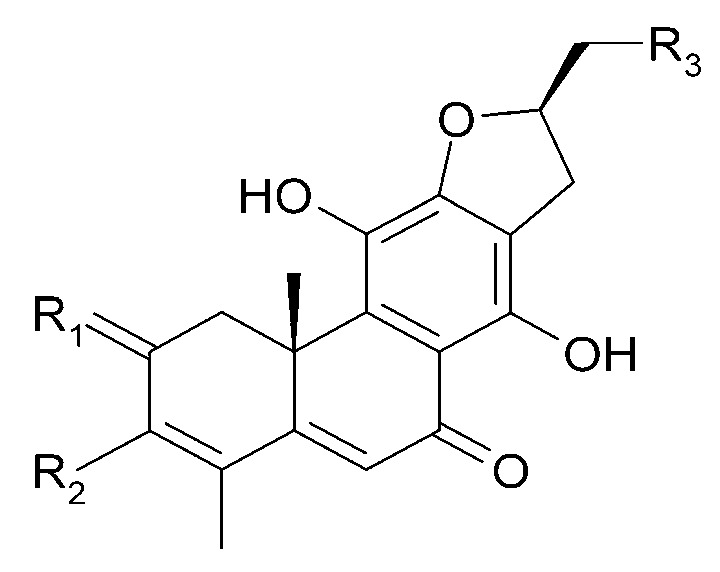
Chemical structures of diterpenoids present in *C. trichotomum*, part 3.

**Figure 13:**
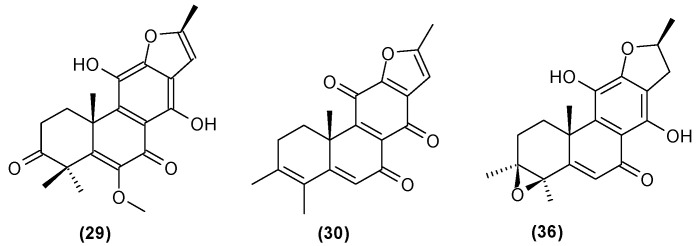
Chemical structures of diterpenoids present in *C. trichotomum*, part 4.

**Figure 14:**
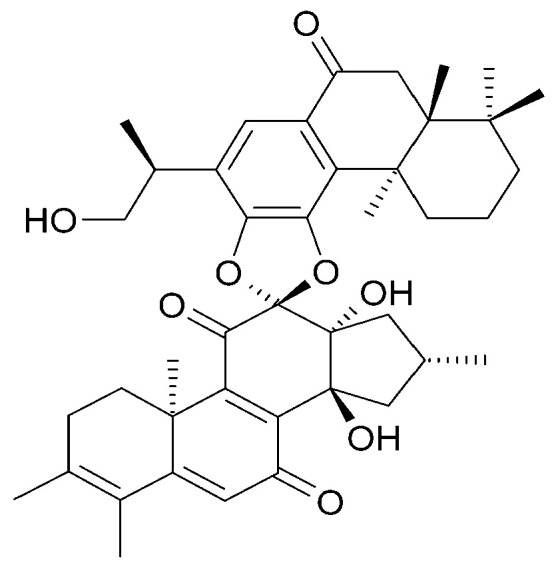
Chemical structures of diterpenoids present in *C. trichotomum*, part 5.

The roots of *C. trichotomum* have proven to be a rich source of abietane diterpenoids. The first report concerned the isolation of 14 compounds from this group: villosin C (**23**), mandarone E (**24**), formidiol (**25**), uncinatone (**26**), teuvincenone E (**27**) and F (**28**), 12,16-epoxy-11,14-dihydroxy-6-methoxy-17(15→16)-abeo-abieta-5,8,11,13,15-pentaene-3,7-dione (**29**), 12,16-epoxy-17(15→16),18(4→3)-diabeo-abieta-3,5,8,12,15-pentaene-7,11,14-trione (trichotomone H) (**30**), and described for the first time: 6-methoxyvillosin C (**31**), 18-hydroxy-6-methoxyvillosin C (**32**), (10R,16S)-12,16-epoxy-11,14-dihydroxy-6-methoxy-17(15→16)-abeo-abieta-5,8,11,13-tetraene-3,7-dione (**33**), (10R,16R)-12,16-epoxy-11,14,17-trihydroxy-17(15→16),18(4→3)-diabeo-abieta-3,5,8,11,13-pentaene-2,7-dione (**34**), (10R,16S)-12,16-epoxy-11,14-dihydroxy-18-oxo-17(15→16),18(4→3)-diabeo-abieta-3,5,8,11,13-pentaene-7-one (trichotomone D) (**35**), (3S,4R,10R,16S)-3,4:12,16-diepoxy-11,14-dihydroxy-17(15→16),18(4→3)-diabeo-abieta-5,8,11,13-tetraene-7-one (trichotomon F) (**36**) (Figure 10, Figure 11, Figure 12 and Figure 13) [27]. Continued this research led to the isolation of the dimeric diterpene trichotomone (**37**) (Figure 14) [28].

(**23**) villosin CR_1_ = 2H;R_2_ = H;R_3_ = OH;R_4_ = OH(**31**) 6-methoxyvillosin CR_1_ = 2H;R_2_ = H;R_3_ = OCH3;R_4_ = OH(**32**) 18-hydroxy-6-methoxyvillosin CR_1_ = 2H;R_2_ = OH;R_3_ = OCH3;R_4_ = OH(**33**) (10R,16S)-12,16-epoxy-11,14-dihydroxy-6-methoxy-17(15→16)-abeo-abieta-5,8,11,13-tetraene-3,7-dioneR_1_ = O;R_2_ = H;R_3_ = OCH3;R_4_ = H

(**24**) mandarone ER_1_ = 2H;R_2_ = CH_3_(**25**) formidiolR_1_ = 2H;R_2_ = COOCH_3_(**28**) teuvincenone FR_1_ = O;R_2_ = CH_3_

(**26**) uncinatoneR_1_ = 2H;R_2_ = CH_3_;R_3_ = H(**27**) teuvincenone ER_1_ = 2O;R_2_ = CH_3_;R_3_ = H(**34**) (10R,16R)-12,16-epoxy-11,14,17-trihydroxy-17(15→16),18(4→3)-diabeo-abieta-3,5,8,11,13-pentaene-2,7-dioneR_1_ = O;R_2_ = CH_3_;R_3_ = OH(**35**) (3S,4R,10R,16S)-3,4:12,16-diepoxy-11,14-dihydroxy-17(15→16),18(4→3)-diabeo-abieta-5,8,11,13-tetraene-7-oneR_1_ = 2H;R_2_ = CHO;R_3_ = H

(**29**) 12,16-epoxy-11,14-dihydroxy-6-methoxy-17(15→16)-abeo-abieta-5,8,11,13,15-pentaene-3,7-dione
(**30**) 12,16-epoxy-17(15→16),18(4→3)-diabeo-abieta-3,5,8,12,15-pentaene-7,11,14-trione
(**36**) (3S,4R,10R,16S)-3,4:12,16-diepoxy-11,14-dihydroxy-17(15→16),18(4→3)-diabeo-abieta-5,8,11,13-tetraene-7-one

(**37**) trichotomone

**Figure 15:**
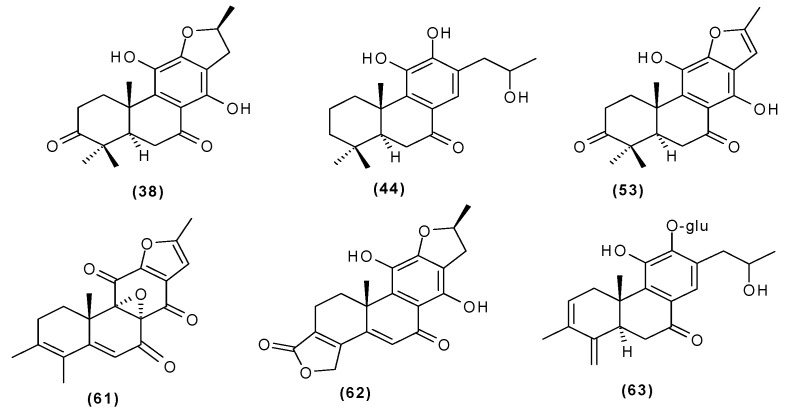
Chemical structures of diterpenoids present in *C. trichotomum*, part 6.

**Figure 16:**
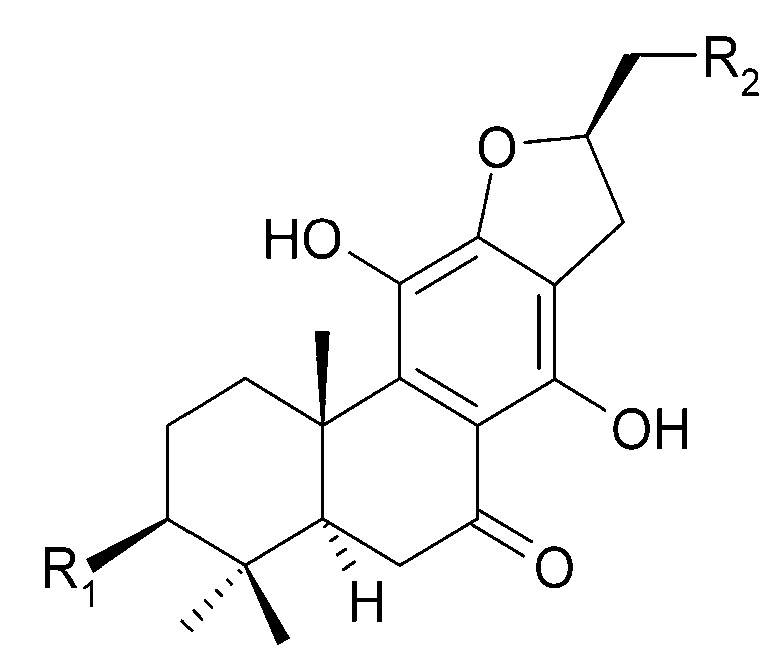
Chemical structures of diterpenoids present in *C. trichotomum*, part 7.

**Figure 17:**
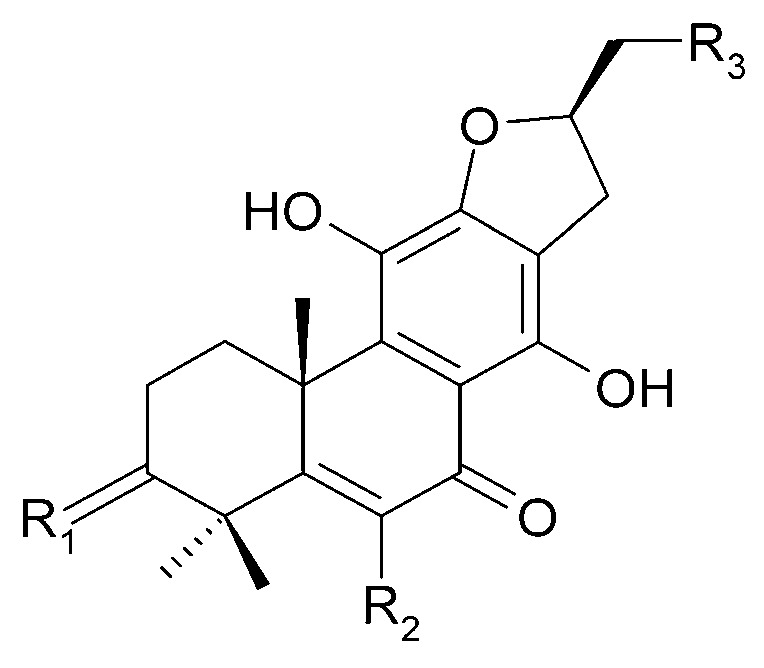
Chemical structures of diterpenoids present in *C. trichotomum*, part 8.

**Figure 18:**
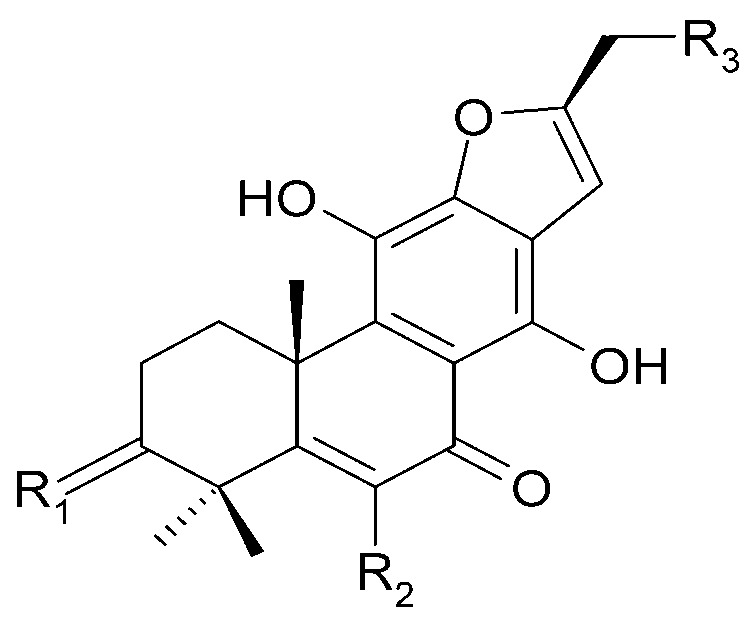
Chemical structures of diterpenoids present in *C. trichotomum*, part 9.

**Figure 19:**
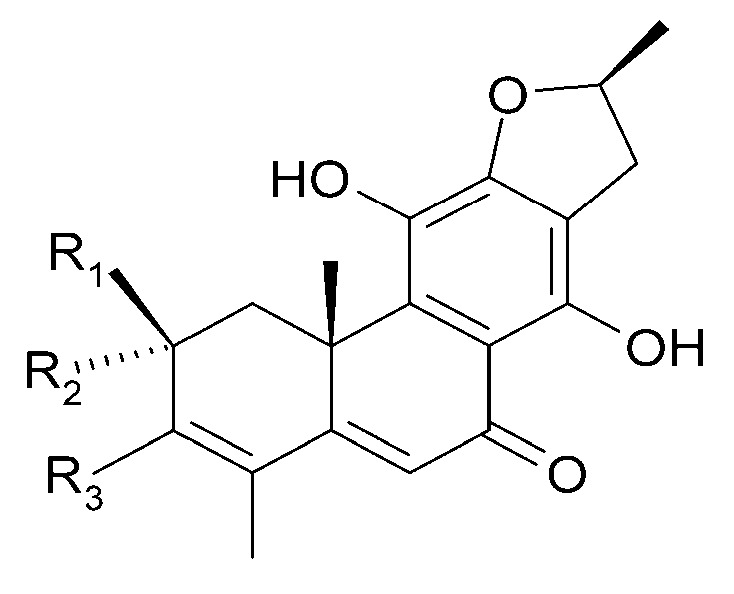
Chemical structures of diterpenoids present in *C. trichotomum*, part 10.

**Figure 20:**
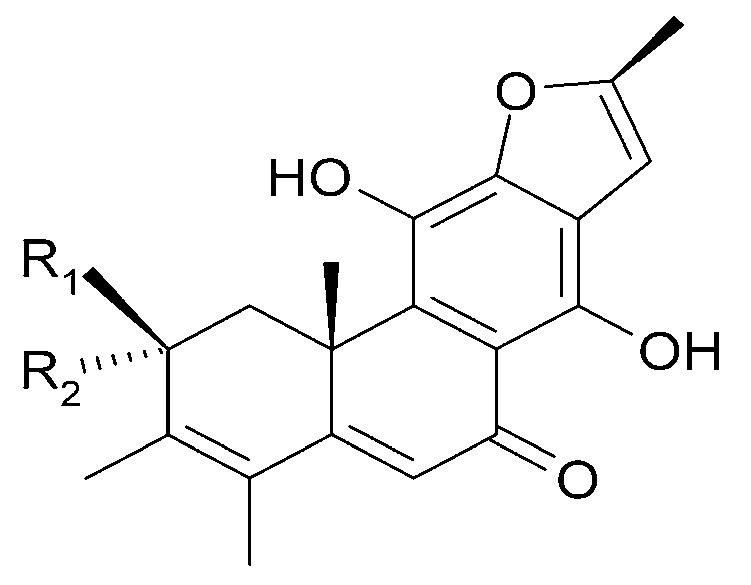
Chemical structures of diterpenoids present in *C. trichotomum*, part 11.

**Figure 21:**
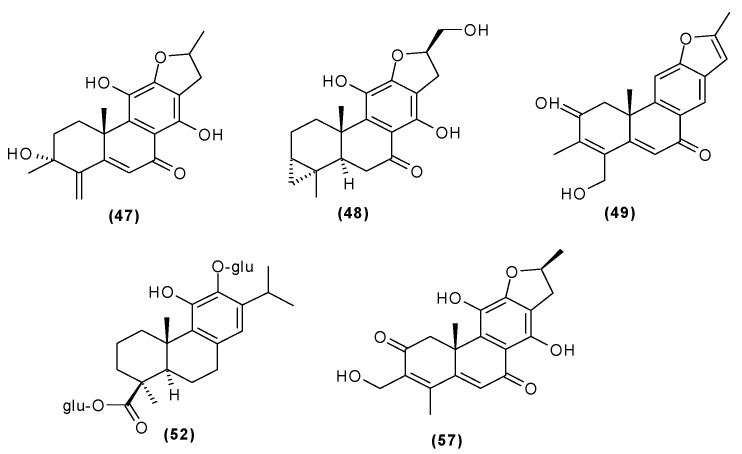
Chemical structures of diterpenoids present in *C. trichotomum*, part 12.

**Figure 22:**
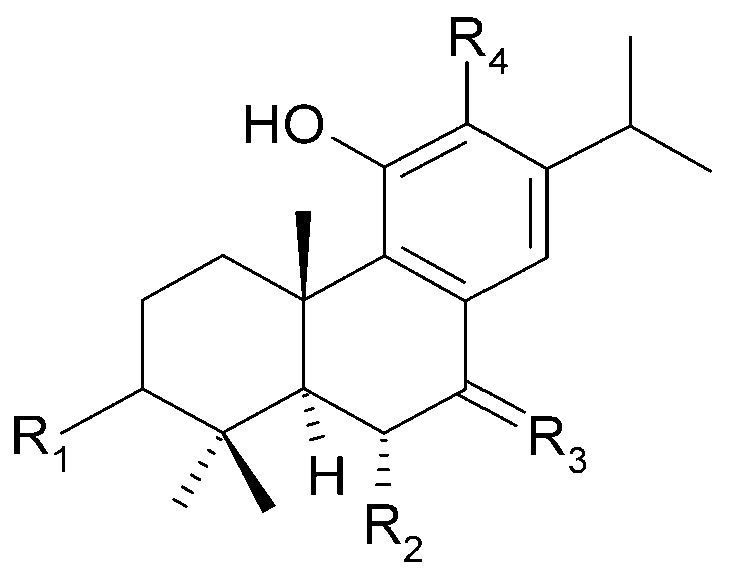
Chemical structures of diterpenoids present in *C. trichotomum*, part 13.

**Figure 23:**
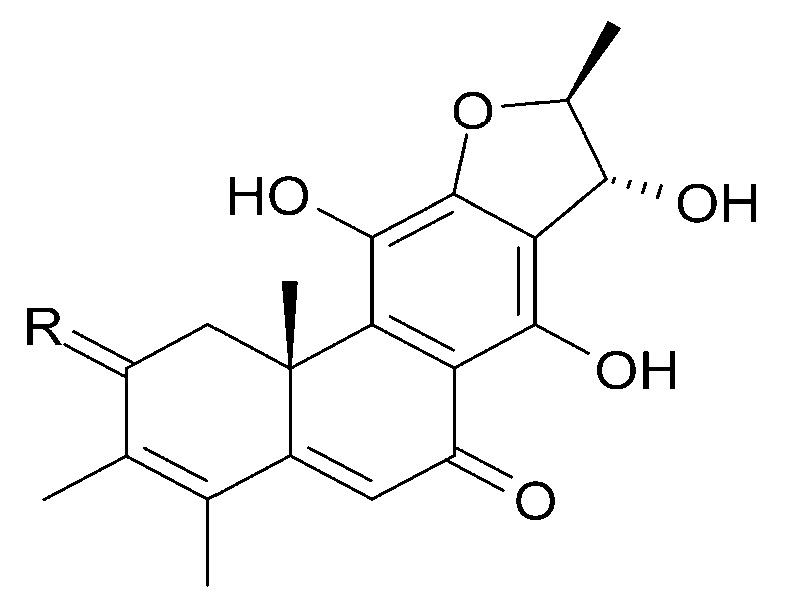
Chemical structures of diterpenoids present in *C. trichotomum*, part 14.

Other abietane diterpenoids were isolated from the roots in 2018 [29]. In addition to those previously described in the roots or stems of *C. trichotomum*, the following were detected: teuvincenone G (**38**), villosin B (**39**), cyrtophyllone A (**40**), 15-dehydro-17-hydroxycyrtophyllone A (**41**), caryopincaolides E (**42**), F (**43**), G (**44**), I (**45**), J (**46**), caryopterisoid C (**47**), kaichianone B (**48**), 19-hydroxyteuvincenone F (**49**), demethylcryptojaponol (50), 6*β*-hydroxydemethylcryptojaponol (**51**), and 12,19-di-*O*-*β*-D-glucopyranosyl-11-hydroxyabieta-8,11,13-trien-19-one (**52**) (Figure 15, Figure 16, Figure 17, Figure 18, Figure 19, Figure 20, Figure 21 and Figure 22). In addition, 12 new compounds were identified and described for the first time: 15,16-dehydroteuvincenone G (**53**), 3-dihydroteuvincenone G (**54**), 17-hydroxymandarone B (**55**), 15,16-dihydroformidiol (**56**), 18-hydroxyteuvincenone E (**57**), 2*α*-hydrocaryopincaolide F (**58**), 15*α*-hydroxyuncinatone (**59**), 15*α*-hydroxyteuvincenone E (**60**), trichotomin A (**61**), trichotomin B (**62**), trichotomside A (**63**), trichotomside B (**64**) (Figure 15, Figure 17, Figure 19, Figure 21, Figure 22 and Figure 23) [29].

(**38**) teucincenone G
(**44**) caryopincaolide G
(**53**) 15,16-dehydroteuvincenone G
(**61**) trichotomin A
(**62**) trichotomin B
(**63**) trichotomside A

(**39**) villosin BR_1_ = H;R_2_ = OH(**54**) 3-dihydroteuvincenone GR_1_ = OH;R_2_ = H

(**40**) cyrtophyllone AR_1_ = 2H;R_2_ = OCH_3_;R_3_ = H(**55**) 17-hydroxymandarone BR_1_ = 2H;R_2_ = H;R_3_ = OH(**66**) teuvincenone AR_1_ = O;R_2_ = OH;R_3_ = H(**67**) teuvincenone BR_1_ = 2H; R_2_ = OH;R_3_ = H

(**41**) 15-dehydro-17-hydroxycyrtophyllone AR_1_ = 2H;R_2_ = OCH_3_;R_3_ = OH(**68**) teuvincenone HR_1_ = O;R_2_ = OH;R_3_ = H

(**42**) caryopincaolide ER_1_ = H;R_2_ = H;R_3_ = CH_2_OH(**43**) caryopincaolide FR_1_ = H;R_2_ = OH;R_3_ = CH_3_(**56**) 15,16-dihydroformidiolR_1_ = H;R_2_ = H;R_3_ = COOCH_3_(**58**) 2*α*-hydrocaryopincaolide FR_1_ = OH;R_2_ = H;R_3_ = CH_3_

(**45**) caryopincaolide IR_1_ = H;R_2_ = OH(**46**) caryopincaolide JR_1_ = OH;R_2_ = H

(**47**) caryopterisoid C
(**48**) kaichianone B
(**49**) 19-hydroxyteuvincenone F
(**52**) 12,19-di-*O*-*β*-D-glucopyranosyl-11-hydroxyabieta-8,11,13-trien-19-one
(**57**) 18-hydroxyteuvincenone E

(**50**) demethylcryptojaponolR_1_ = H;R_2_ = H;R_3_ = O;R_4_ = OH(**51**) 6*β*-hydroxydemethylcryptojaponolR_1_ = H;R_2_ = OH;R_3_ = O;R_4_ = OH(**64**) trichotomside BR_1_ = O-glucose;R_2_ = H;R_3_ = 2H;R_4_ = O-glucose

(**59**) 15*α*-hydroxyuncinatoneR = 2H(**60**) 15*α*-hydroxyteuvincenone E R = O

**Figure 24:**
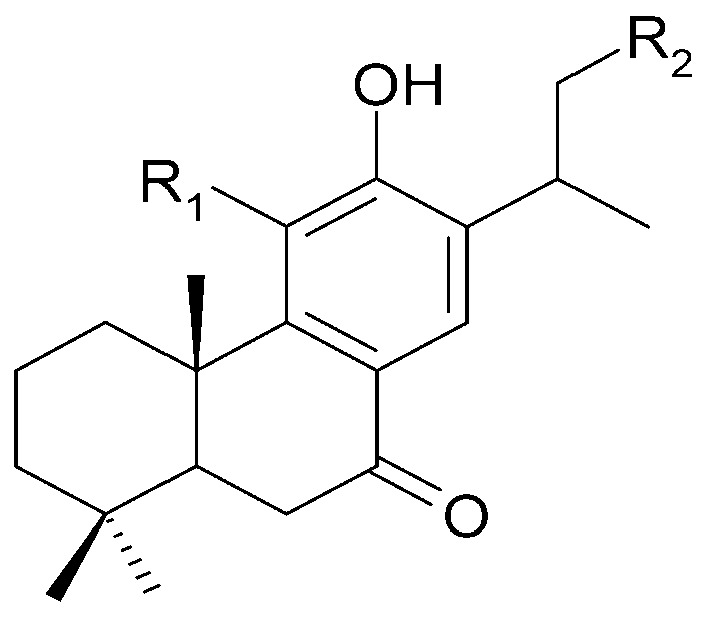
Chemical structures of diterpenoids present in *C. trichotomum*, part 15.

**Figure 25:**
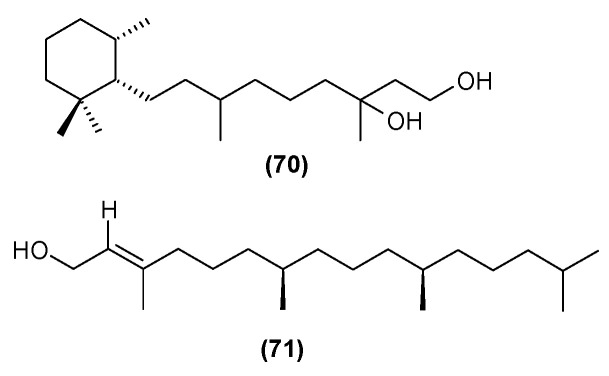
Chemical structures of diterpenoids present in *C. trichotomum*, part 16.

Seven abietane diterpenoids were isolated from the stems of *C. trichotomum*. In addition to those previously described in this species, teuvincenone F (**28**) and uncinatone (**26**), the presence of sugiol (**65**) (Figure 24), teuvincenone A (**66**), B (**67**) (Figure 17), and H (**68**) (Figure 18), as well as cyrtophyllone B (**69**) (Figure 24) were reported for the first time [8]. Additionally, the presence of viridiol B (**70**) and phytol (**71**) were also noted in the leaves of the plant (Figure 25) [15]. 

(**65**) sugiolR_1_ = H;R_2_ = H(**69**) cyrtophyllone BR_1_ = OH;R_2_ = OH

(**70**) viridiol B
(**71**) phytol

**Figure 26:**
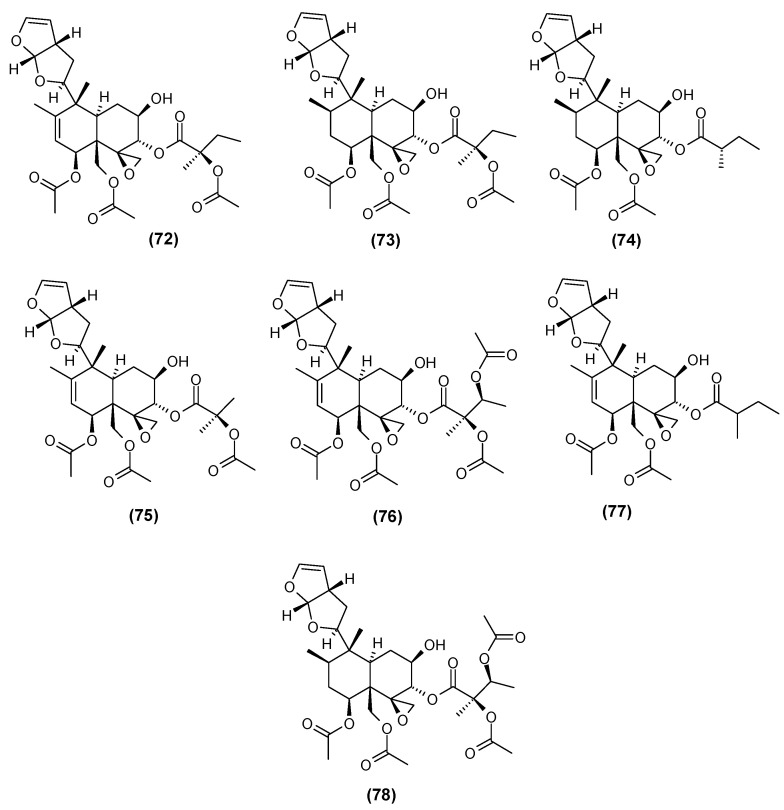
Chemical structures of diterpenoids present in *C. trichotomum*, part 17.

Another group of diterpenes present in *C. trichotomum* leaves are clerodendrins (Figure 26). The first one detected in the leaves of this species was clerodendrin A (**72**) [30]; two more, B (**73**) and D (**74**), were described in 1989 [31], and another four (E, F, G and H, respectively, **75**, **76**, **77**, **78**) nine years later [32].

(**72**) clerodendrin A
(**73**) clerodendrin B
(**74**) clerodendrin D
(**75**) clerodendrin E
(**76**) clerodendrin F
(**77**) clerodendrin G
(**78**) clerodendrin H.

Isolation of numerous abietane diterpenoids from *C. trichotomum*, chemotaxonomic markers of the Lamiaceae, support the reclassification of this genus from Verbanaceae to Lamiaceae (Figure 2).

#### 5.2.2. Triterpenoids

**Figure 27:**
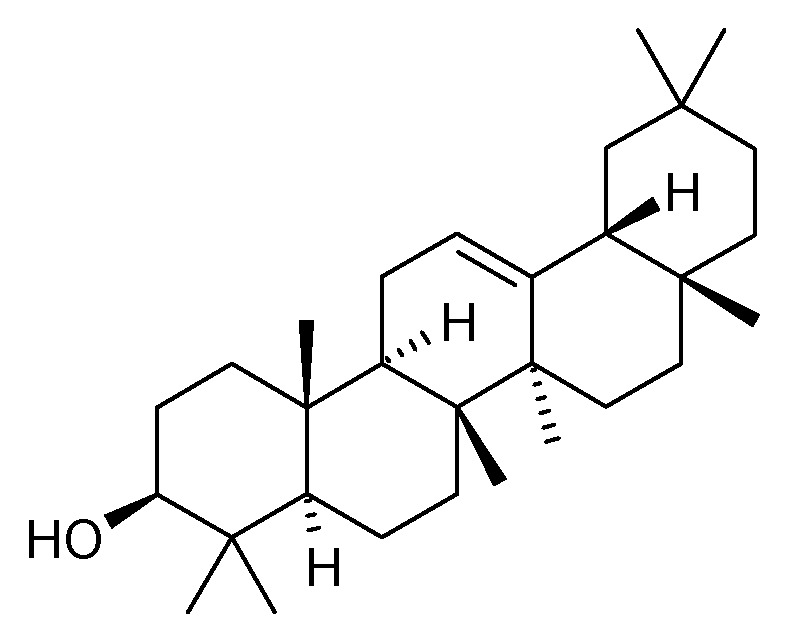
Chemical structures of triterpenoids present in *C. trichotomum*, part 1.

**Figure 28:**
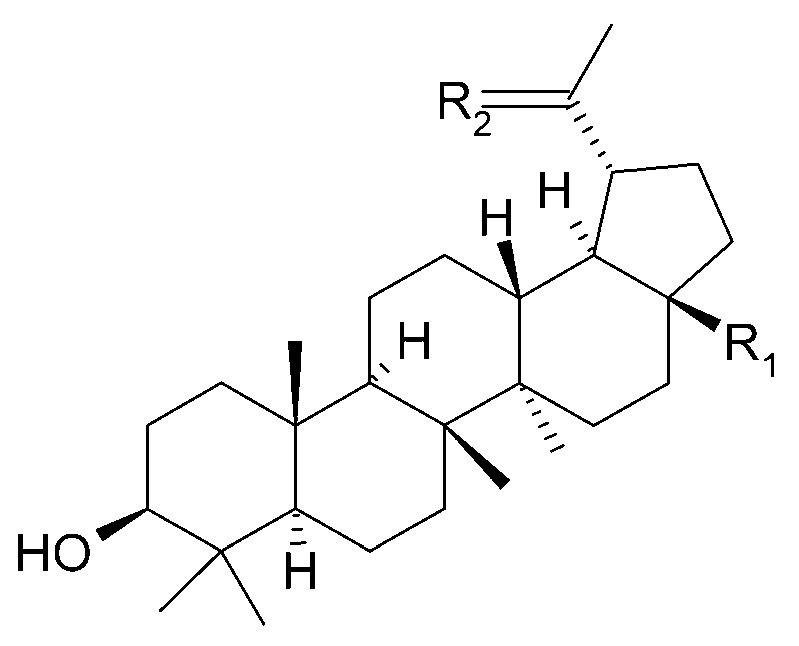
Chemical structures of triterpenoids present in *C. trichotomum*, part 2.

**Figure 29:**
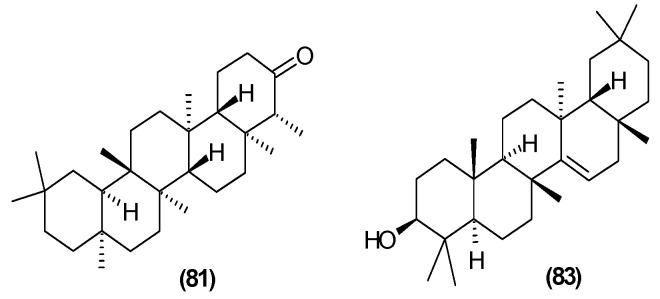
Chemical structures of triterpenoids present in *C. trichotomum*, part 3.

**Figure 30:**
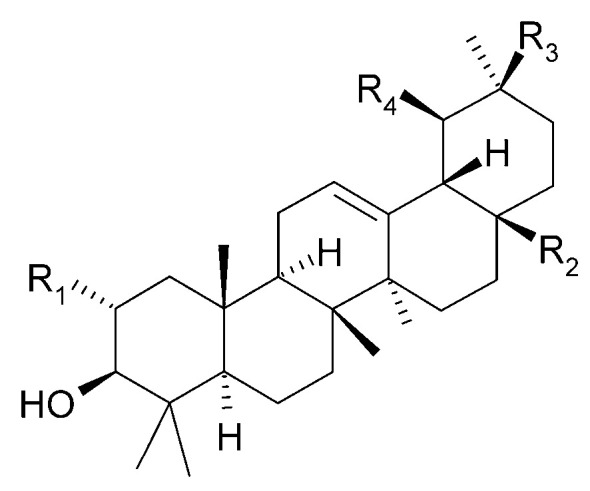
Chemical structures of triterpenoids present in *C. trichotomum*, part 4.

The presence of *β*-amyrin (**79**) was revealed in the methyl chloride fraction of the methanol extract from the stem of *C. trichotomum* (Figure 27) [33], whereas lupeol (**80**), friedelin (**81**), betulinic acid (**82**), and taraxerol (**83**) were found in the petroleum ether extract of leaves of the plant (Figure 28 and Figure 29) [34]. A year later, aside from betulinic acid (**82**) and lupeol (**80**), Xu et al. [15] identified triterpenoids such as 3*β*-hydroxy-30-norlupan-20-one (**84**), oleanolic (**85**) and ursolic (**86**) aldehydes, maslinic acid (**87**), and corosolic acid (**88**) (Figure 28 and Figure 30) from the leaves, with the latter compound being reported in the *Clerodendrum* genus for the first time. Their structures were determined based on comparing NMR (nuclear magnetic resonance) and ESI-MS (electrospray ionization mass spectrometry) profiles with data from available literature sources.

(**79**) *β*-amyrin

(**80**) lupeolR_1_ = CH_3_;R_2_ = CH_2_(**82**) betulinic acidR_1_ = COOH;R_2_ = CH_2_(**84**) 3*β*-hydroxy-30-norlupan-20-oneR_1_ = CH_3_;R_2_ = O

(**81**) friedelin
(**83**) taraxerol

(**85**) oleanolic aldehydeR_1_ = H;R_2_ = CHO;R_3_ = CH_3_;R_4_ = H(**86**) ursolic aldehydeR_1_ = H;R_2_ = CHO;R_3_ = H;R_4_ = CH_3_(**87**) maslinic acidR_1_ = OH;R_2_ = COOH;R_3_ = CH_3_;R_4_ = H(**88**) corosolic acidR_1_ = OH;R_2_ = COOH;R_3_ = H;R_4_ = CH_3_

### 5.3. Anthraquinones

**Figure 31:**
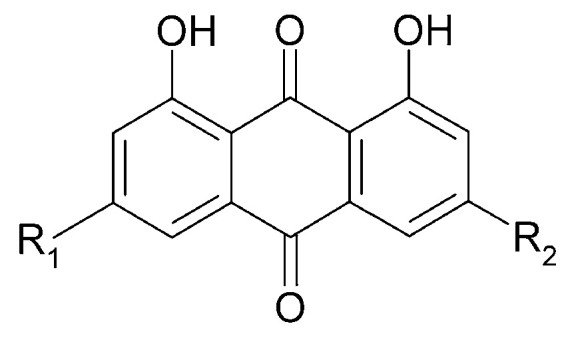
Chemical structures of anthraquinones present in *C. trichotomum*.

In 2014, three anthraquinone compounds were isolated from *C. trichotomum*: aloeemodin (**89**), emodin (**90**), and chrysophanol (**91**) (Figure 31). This was the first report of the presence of these compounds not only in *C. trichotomum*, but also in the Lamiaceae family [8].

(**89**) aloeemodinR_1_ = H;R_2_ = CH_2_OH(**90**) emodinR_1_ = CH_3_;R_2_ = OH(**91**) chrysophanolR_1_ = H;R_2_ = CH_3_

### 5.4. Cyclohexylethanoids

**Figure 32:**
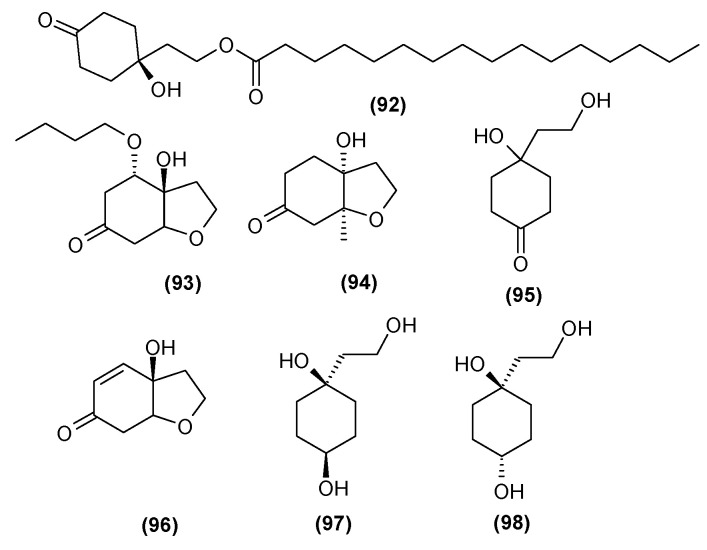
Chemical structures of cyclohexylethanoids present in *C. trichotomum*.

In 2014, Xu et al. [35] isolated seven compounds belonging to the group of cyclohexylethanoids from the leaves of *C. trichotomum*. Two of them were described for the first time: 1-hydroxy-1-(8-palmitoyloxyethyl) cyclohexanone (**92**), occurring as colorless needles, and 5-*O*-butyl cleroindin D (**93**), appearing as a colorless oily liquid. The remaining five compounds from this group, viz. cleroindin C (**94**), cleroindin B (**95**), rengyolone (**96**), rengyol (**97**), and isorengylon (**98**), had been described earlier, with some being present in other *Clerodendrum* species [35,36]. These compounds are illustrated in Figure 32.

(**92**) 1-hydroxy-1-(8-palmitoyloxyethyl)cyclohexanone
(**93**) 5-*O*-butyl cleroindin
(**94**) cleroindin C
(**95**) cleroindin B
(**96**) rengyolone
(**97**) rengyol
(**98**) isorengyol

### 5.5. Steroids

**Figure 33:**
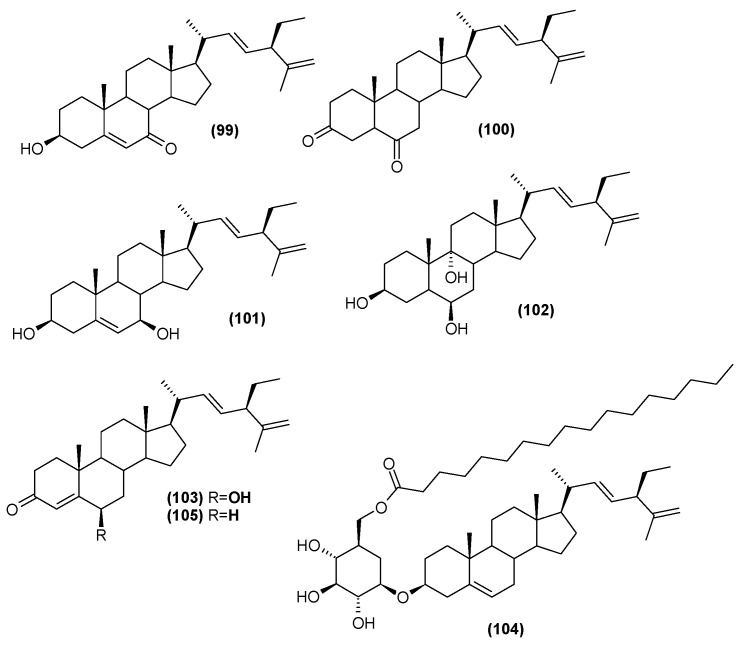
Chemical structures of steroids present in *C. trichotomum*, part 1.

**Figure 34:**
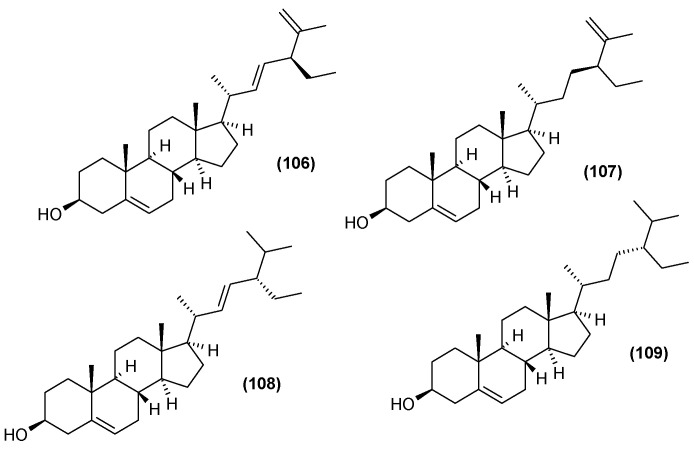
Chemical structures of steroids present in *C. trichotomum*, part 2.

The leaf extract of *C. trichotomum* was also found to include steroids. Seven were isolated in 2013, five of which were described for the first time [37]: (20R,22E,24R)-3*β*-hydroxy-stigmasta-5,22,25-trien-7-one (**99**), (20R,22E,24R)-stigmasta-22,25-dien-3,6-dione (**100**), (20R,22E,24R)-stigmasta-5,22,25-trien-3*β*,7*β*-diol (**101**), (20R,22E,24R)-stigmasta-22,25-dien-3*β*,6*β*,9*α*-triol (**102**), (20R,22E,24R)-6*β*-hydroxy-stigmasta-4,22,25-trien-3-one (**103**). The two previously-known compounds were 22-dehydroclerosterol 3*β*-*O*-*β*-D-(6′-*O*-margaroyl)-glucopyranoside (**104**), and (22E,24R)-stigmasta-4,22,25-trien-3-one (**105**) (Figure 33). In 2014, four other sterols were isolated from the leaves: 22-dehydroclerosterol (**106**), clerosterol (**107**), stigmasterol (**108**), and sitosterol (**109**) (Figure 34) [34].

(**99**) (20R,22E,24R)-3*β*-hydroxy-stigmasta-5,22,25-trien-7-one
(**100**) (20R,22E,24R)-stigmasta-22,25-dien-3,6-dione
(**101**) (20R,22E,24R)-stigmasta-5,22,25-trien-3*β*,7*β*-diol
(**102**) (20R,22E,24R)-stigmasta-22,25-dien-3*β*,6*β*,9*α*-triol
(**103**) (20R,22E,24R)-6*β*-hydroxy-stigmasta-4,22,25-trien-3-one
(**104**) 22-dehydroclerosterol 3*β*-*O*-*β*-D-(6′*-O*-margaroyl)-glucopyranoside
(**105**) (22E,24R)-stigmasta-4,22,25-trien-3-one

(**106**) 22-dehydroclerosterol
(**107**) clerosterol
(**108**) stigmasterol
(**109**) sitosterol

### 5.6. Polyketones

**Figure 35:**
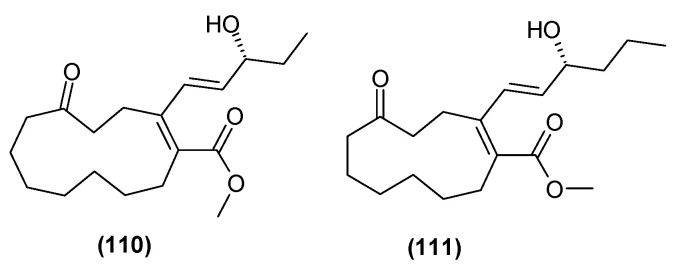
Chemical structures of polyketones present in *C. trichotomum*.

Relatively recently, in 2022, the isolation of two new polyketones from the leaves and branches of *C. trichotomum* were described: clerodendruketone A (**110**) and clerodendruketone B (**111**) (Figure 35) [26].

(**110**) clerodendruketone A
(**111**) clerodendruketone B

### 5.7. Alkaloids

**Figure 36:**
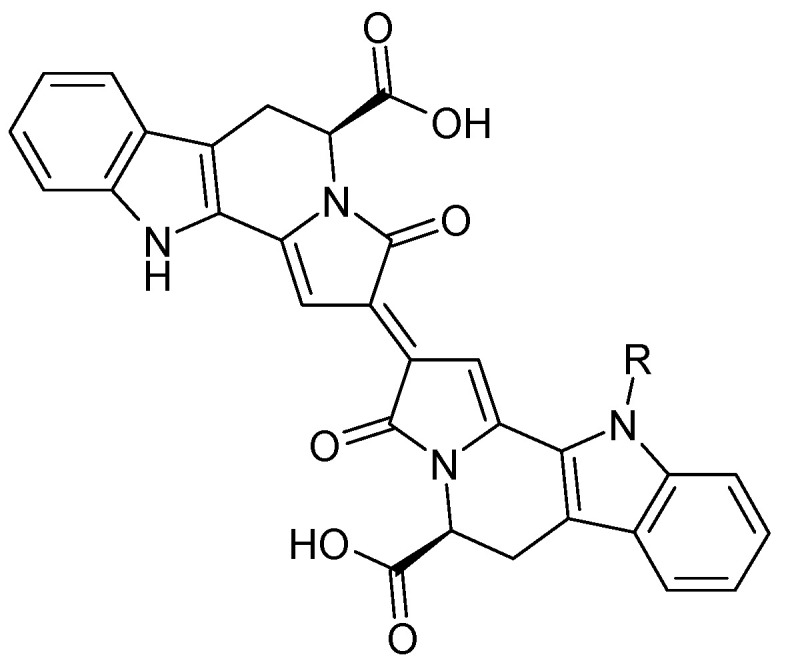
Chemical structures of alkaloids present in *C. trichotomum*, part 1.

The first compound isolated from *C. trichotomum* was the blue trichotomine (**112**), present in the fruits of this species, along with its sugar derivative, trichotomine G1 (**113**) (Figure 36). Earlier attempts to isolate this pigment took place in the 1940s but were unsuccessful [38]. Several years later, indolizino [8,7-b] indole 5-carboxylic acids were obtained, which are precursors to trichotomines [39].

(**112**) trichotomineR = H(**113**) trichotomine G1R = glucose

**Figure 37:**
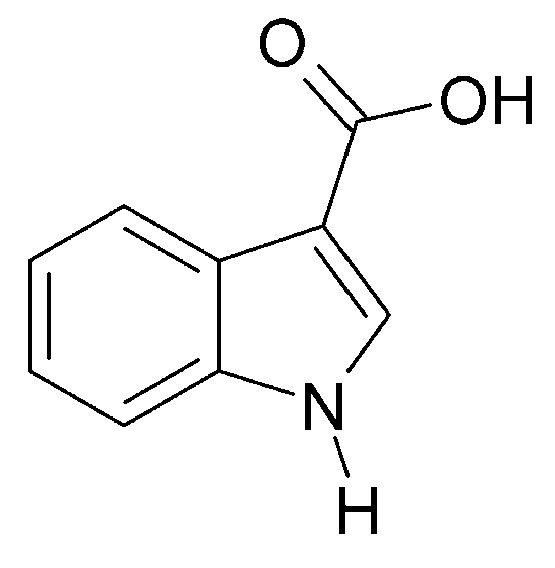
Chemical structures of alkaloids present in *C. trichotomum*, part 2.

Petroleum ether extract of *C. trichotomum* leaves was found to contain an alkaloid with a structure that was confirmed by spectroscopic analysis as 1H-indole-3-carboxylic acid (**114**) (Figure 37). In the study, this compound was obtained for the first time from the *Clerodendrum* genus [34].

(**114**) 1H-indole-3-carboxylic acid

### 5.8. Other Compounds

**Figure 38:**
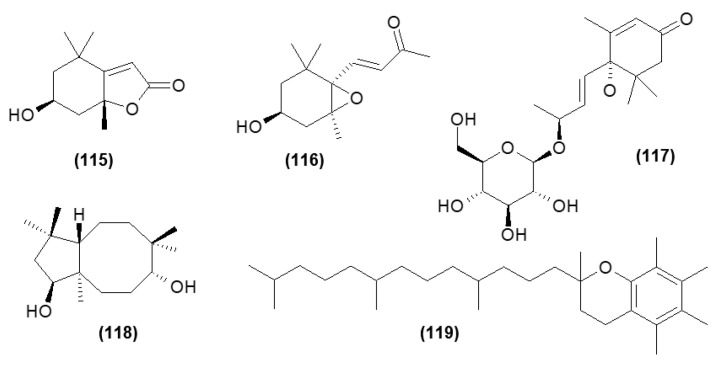
Chemical structures of other compounds present in *C. trichotomum*.

Moreover, the heterocyclic compounds such as loliolide (**115**), annuionone D (**116**), monoterpene glucoside—corchoionoside C (**117**), sesquiterpene: clovane-2,9-diol (**118**), and α-tocopherol (**119**) have also been isolated from the *C. trichotomum* leaf [15]. Their structures are shown in Figure 38.

(**115**) loliolide
(**116**) annuionone D
(**117**) corchoionoside C
(**118**) clovane-2,9-diol
(**119**) *α*-tocopherol

## 6. Biological Activity of *C. trichotomum*

In the last decade, some attempts have been made to confirm the biological activity of *C. trichotomum* and identify the metabolites responsible for it.

### 6.1. Anti-Inflammatory Activity

The main application of *C. trichotomum* is connected to its anti-inflammatory potential. Research based on murine RAW 264.7 macrophage cell lines and mouse and rat models found that inflammation induced by carrageenan in rat paw was reduced by 23% with the application of a 60% methanol leaf extract fraction of *C. trichotomum* at a dose of 1 mg/kg. The same product reduced capillary permeability induced by intraperitoneal injection of acetic acid in mice by 47% and inhibited prostaglandin E2 (PGE2) production in RAW 264.7 macrophages treated with lipopolysaccharide (LPS). All these effects were at least as strong as those obtained in the tests for indomethacin, indicating significant anti-inflammatory activity [3]. 

A subsequent report found that the methanol fraction of the leaf extract of *C. trichotomum* significantly reduced the amount of PGE2 in a dose-dependent manner, with isoacteoside (1) and acteoside (7) identified as the main compounds exhibiting this activity. Further experiments included tests on vascular permeability in mice and a carrageenan-induced edema model in rats. The 80% methanol fraction reduced dye leakage with a maximum inhibitory activity of 47.0%, with similar inhibition reported for pure acteoside (46.5%). In the carrageenan-induced edema model, the 80% methanol fraction reduced the frequency of edema occurrence by 59.5%, and acteoside by 63.8% [22].

Research on the anti-inflammatory effects of *C. trichotomum* was also conducted by a Korean research team at Jeju National University. The ethyl acetate fraction obtained from a 70% ethanol extract of the leaves effectively inhibited nitric oxide (NO) production in LPS-stimulated RAW 264.7 cells, with an IC_50_ of 18.87 μg/mL. Application of this fraction (100 μg/mL) also effectively reduced tumor necrosis factor *α* (TNF-*α*) and interleukin-6 (IL-6) levels, as well as the expression of inducible nitric oxide synthase (iNOS) and cyclooxygenase-2 (COX-2) in RAW 264 cells stimulated by LPS. These results indicate the promising anti-inflammatory potential of the product [4].

A mixture of six plant ferments, including one obtained from *C. trichotomum*, was administered to mice with allergic rhinitis induced by egg albumin. A product, fermented for 4 days, reduced allergic symptoms with a similar effectiveness to cetirizine. It also significantly reduced the levels of histamine and immunoglobulin E in the blood, which were released during contact with the allergen. Additionally, the ferment mixture containing *C. trichotomum* was found to inhibit the activity of COX and 5-LO enzymes, which are generators of pro-inflammatory mediators [40].

### 6.2. Antioxidant Activity

Several studies describe the evaluation of the antioxidant activity of methanolic extract of *C. trichotomum* and its constituent compounds. The first report examined the antioxidant activity of jionoside D (**4**) based on its ability to scavenge intracellular free radicals and neutralize DPPH (1,1-diphenyl-2-picrylhydrazyl) radicals. At a concentration of 10 mg/mL, jionoside D reduced intracellular ROS levels by 86%; the result was comparable to the control (N-acetylcysteine). It also neutralized approximately 55% of DPPH free radicals at a concentration of 10 mg/mL. Additionally, jionoside D inhibited lipid peroxidation in hamster lung fibroblasts treated with hydrogen peroxide (H_2_O_2_), increased cell viability, prevented H_2_O_2_-induced apoptosis, and enhanced the activity of the enzymes superoxide dismutase (SOD) and catalase (CAT), protecting against oxidative stress [5].

The antioxidant properties of trichotomoside (**8**) were also examined. It was found to effectively scavenge intracellular reactive oxygen species (ROS) and DPPH radicals, and it increased the viability of cells exposed to γ-radiation and H_2_O_2_, with greater efficacy observed against the former. Like jionoside D, trichotomoside scavenged intracellular radicals more effectively than DPPH radicals, suggesting that it acts not only as a conventional radical scavenger but also indirectly stimulates the cellular defense system [17].

Another study revealed that the 80% methanol fraction obtained from *C. trichotomum* leaves, as well as its component compounds acteoside (**1**), isoacteoside (**7**), and decaffeoylacteoside (**9**), exhibited significant DPPH radical scavenging activity and inhibited the oxidation of LDL (low-density lipoproteins) induced by Cu^2+^. The IC_50_ values for the methanol fraction in these tests were 156.7 µg/mL and 41.83 µg/mL, respectively, and for acteoside, the most potent pure compounds were 19.9 µg/mL and 63.3 µg/mL, respectively [22].

In another experiment, *C. trichotomum* leaves were extracted using 70% ethanol, and the obtained extract was fractionated using hexane, chloroform (CHCl_3_), ethyl acetate (EtOAc), and n-butanol (BuOH). The individual fractions were then analyzed for their antioxidant properties. The EtOAc and BuOH fractions exhibited strong DPPH and ABTS (2,2′-azino-bis[3-ethylbenzothiazoline-6-sulfonic acid]) scavenging activity, with IC_50_ values of 72.05 and 52.12 μg/mL for the DPPH test, and 33.86 and 25.40 μg/mL for the ABTS assay, respectively [4].

The studies also showed that the crude, unfractionated methanolic extract of *C. trichotomum* exhibited DPPH radical scavenging activity, with an IC_50_ value of 33 µg/mL and iron ion reduction at a level of 1045 mM Fe(II)/g dry weight [41]. DPPH radical scavenging activity was also examined for recently-isolated polyketones and lignans. It was found that all lignans of *C. trichotomum* (**19**–**22**) possessed moderate antioxidant activity, with IC_50_ values ranging from 53.6 µM to 68.9 µM, whereas the polyketones, clerodendruketone A (**110**) and B (**111**), did not [26].

### 6.3. Anticancer Activity

One of the most important areas of research in contemporary medicine is the search for compounds with anticancer activity. Polyphenolic compounds obtained from the bark and leaves of *C. trichotomum* and their derivatives were evaluated for cytotoxicity on three cancer cell lines: MK-1 (human gastric adenocarcinoma), HeLa (human cervical cancer), and B16F10 (mouse melanoma). The strongest antiproliferative activity was observed for acteoside (**1**) and isoacteoside (**7**) (with IC_50_ values ranging from 8 to 66 µM). Therefore, the researchers suggest that the key element of the structure responsible for this activity may be the 3,4-dihydroxyphenethyl group, rather than the earlier suggested caffeoyl moiety [16]. Cytotoxic properties against HeLa cell lines were also confirmed for two steroids isolated from *C. trichotomum* leaves: (20R,22E,24R)-3*β*-hydroxy-stigmasta-5,22,25-trien-7-one (**99**) and (20R,22E,24R)-stigmasta-5,22,25-trien-3*β*,7*β*-diol (**101**), with IC_50_ values of 35.67 mg/mL and 28.92 mg/mL, respectively [37].

Wu et al. evaluated the cytotoxicity of the diterpenes isolated from the roots of *C. trichotomum* against five human cancer cell lines: BGC-823 and KE-97 (gastric cancer), Huh-7 (liver cancer), KB (nasopharyngeal cancer), and Jurkat (acute T-cell lymphoblastic leukemia). Cytotoxic activity was observed for trichotomone D (**35**), F (**36**), and H (**30**), uncinatone (**26**), mandarone E (**24**), and teuvincenone E (**27**), with IC_50_ values ranging from 0.83 to 50.99 μM [27]. Among these, teuvincenone E exhibited the strongest activity against these five cell lines, with IC_50_ values of 3.95 µM, 5.37 µM, 1.18 µM, 1.27 µM, and 0.83 µM, respectively. Based on the collected data, the authors suggest that the cytotoxic activity of this group of compounds derived from the rearranged A-ring and an intact 2-methyl-2-dihydrofuran moiety [27].

In addition, the dimeric diterpene trichotomone (**37**) also demonstrated cytotoxic activity against several human cell lines (viz. A549–lung cancer, Jurkat–acute T-cell lymphoblastic leukemia, BGC-823–gastric cancer, and 293T WT–kidney cells), with IC_50_ values ranging from 7.51 to 19.38 µM [28].

### 6.4. Antiviral Activity

Studies have investigated the potential of the phenylpropanoids of *C. trichotomum* to inhibit HIV-1 integrase (human immunodeficiency virus) [21]. The strongest activity was reported for acteoside (**1**) and isoacteoside (**7**), with IC_50_ values of 7.8 µM and 13.7 µM. While significant inhibition against HIV-1 integrase was also exhibited by leucosceptoside A (**2**), plantainoside C (**3**), and jionoside D (**4**) (29.4–60.9 µM), no such activity was observed for martynoside (**5**) or isomartynoside (**6**). These results suggest that for phenylpropanoid glycosides to inhibit HIV-1 integrase, they require two catechol groups, and the activity of these compounds decreases as the number of methoxy groups in the aromatic ring increases. However, the location of the feruloyl or caffeoyl substituent in the sugar part does not significantly influence the activity [21].

Chathuranga et al. [42] investigated aqueous extracts from *C. trichotomum* and its main component acteoside (**1**) for their activity against RSV (respiratory syncytial virus). It was found that both products limited virus replication and the death of virus-infected cells, with IC_50_ values of 27.95 µg/mL for the extract and 15.64 µg/mL for acteoside. The extract from *C. trichotomum* leaves and acteoside reduced the level of viral mRNA and virus protein synthesis. Additionally, in studies on a mouse model, the formation of syncytia was inhibited, preventing the spread of the virus [42].

Moreover, molecular docking studies suggest that taraxerol (**83**), friedelin (**81**), and stigmasterol (**108**) isolated from *C. trichotomum* leaves may have promising anti-SARS-CoV-2 (severe acute respiratory syndrome coronavirus 2) potential [43].

### 6.5. Antibacterial Activity

A study of the antibacterial activity of n-hexane, methylene chloride, ethyl acetate, and n-butanol fractions of *C. trichotomum* extract against *Staphylococcus aureus*, *Escherichia coli*, and *Helicobacter pylori* found the methylene chloride fraction to be active against *H. pylori*; in addition, the compounds isolated from it, 22-dehydroclerosterol (**106**) and *β*-amyrin (**79**), had moderate activity against *S. aureus* and *E. coli* at a concentration of 3.4 mg/mL [36]. In another study, ethanol extract of *C. trichotomum* inhibited the growth of both Gram-positive and Gram-negative bacteria, such as *S. aureus*, *E. coli*, *Proteus vulgaris*, and *Klebsiella pneumoniae* [38].

Furthermore, the antibacterial activity of polyketones isolated from *C. trichotomum* leaves against *Escherichia coli* and *Staphylococcus aureus* was analyzed by turbidimetry. Clerodendruketone A (**110**) at a concentration of 50 µg/mL exhibited a bacteriostatic effect ranging between 30% and 60% against *Escherichia coli*, and between 60% and 80% against *Staphylococcus aureus*. In contrast, for clerodendruketone B (**111**), the values for the two tested microorganisms ranged from 30% to 60% [26].

### 6.6. Antihypertensive Activity

One of the traditional uses of *C. trichotomum* preparations is to lower blood pressure. Oral administration of *C. trichotomum* leaf extract reduced blood pressure in spontaneously hypertensive rats, but not in normotensive animals. Intravenous administration resulted in the dilation of renal vessels, increased urine flow, and sodium excretion leading to blood pressure reduction [6].

Subsequent studies have evaluated the inhibition of angiotensin-converting enzyme (ACE) by phenylpropanoids isolated from the stem of *C. trichotomum*: acteoside (**1**), leucosceptoside A (**2**), martynoside (**5**), isoacteoside (**7**), and isomartynoside (**6**). The following respective IC_50_ values were obtained: 373 µg/mL, 423 µg/mL, 524 µg/mL, 376 µg/mL, 505 µg/mL. This indicates that the strongest effect was demonstrated by acteoside and isoacteoside. Furthermore, it appears that the antihypertensive action of *C. trichotomum* is at least partially due to the inhibitory effect of its phenylpropanoid glycosides on ACE, with this effect increasing with increasing numbers of hydroxyl groups on the aromatic rings [44].

Teas from the leaves and flowers of *C. trichotomum* were orally administered to spontaneously hypertensive rats for eight weeks to estimate their effect on blood pressure and heart rate. After this period, it was found that systolic blood pressure in the rats had decreased by 12.5%, diastolic blood pressure by 44.9%, and heart rate by 24.5%, compared to untreated controls; these differences were all significant [7].

### 6.7. Activity in Metabolic Diseases

The effectiveness of *C. trichotomum* tea in lowering cholesterol levels has been documented. Oral administration for a period of eight weeks reduced body weight, total cholesterol, and triglycerides in rats by between 20 and 30% compared to the control group [7].

Jang et al. [45] evaluated the effect of a 70% methanol extract from *C. trichotomum* leaves on metabolic disorders induced by a high-fructose diet. The extract was administered to rats with water for 16 weeks at a dose of 500 mg/kg body weight. It was found that the *C. trichotomum* extract alleviated the effects of a high-fructose diet by reducing body weight gain and hyperglycemia, and it improved the disruption of serum lipid profiles in rats. Additionally, the product alleviated insulin resistance and liver steatosis by influencing the signaling pathways of AMP-activated protein kinase, peroxisome proliferator-activated receptor *α* (PPAR*α*), and sterol regulatory element-binding protein 1; hence, it may be a promising therapeutic agent against metabolic disorders [45].

The effect of the raw material on hyperuricemia and associated inflammation has also been examined. In the first experiment, *C. trichotomum* leaves were extracted using 70% ethanol, and the obtained extract was fractionated using hexane, chloroform (CHCl_3_), ethyl acetate (EtOAc), and n-butanol (BuOH). The study evaluated the ability of individual fractions to inhibit xanthine oxidase activity, thus reducing uric acid production and protecting against hyperuricemia. It was shown that the strongest inhibitors were the CHCl_3_ (IC_50_ = 4.43 μg/mL) and EtOAc (IC_50_ = 5.69 μg/mL) fractions [4].

In the second study, a leaf extract of *C. trichotomum* at a dose of 400 mg/kg body weight was administered to mice with hyperuricemia induced by potassium oxonate. The supplementation significantly reduced the levels of uric acid and creatinine in the blood while increasing their levels in the urine. Moreover, the extract alleviated inflammation and apoptosis induced by potassium oxonate by increasing the level of anti-apoptotic Bcl-2 and decreasing the level of pro-apoptotic Bax in kidney tissues [46].

### 6.8. Other Activities

It has been found that 7-*O*-glucoside of apigenin (**10b**) isolated from *C. trichotomum* leaves can inhibit reflux esophagitis and gastric inflammation in rats. Administration of the compound reduced the volume of gastric juice, increased gastric pH, and significantly reduced the size of lesions induced by exposure of the gastric mucosa to indomethacin [23].

## 7. Toxicity of *Clerodendrum* Plants

Although no toxicological data currently exists on *C. trichotomum* and its products, some reports exist for other species in the genus, suggesting that they may be safe for use. However, this does not eliminate the need for such testing of *C. trichotomum*.

The leaf and root extracts of *C. infortunatum* given orally for 15 days at 2 and 3 g/kg body weight did not cause changes in body weight or movement patterns in Swiss albino mice compared to controls [47]. Also, biochemical, hematological, and histopathological studies revealed no significant differences to the control. The extracts of this species thus appear safe and non-toxic for animals. In addition, the ethyl acetate, chloroform, ethanolic, and aqueous extracts of *C. inerme* did not demonstrate any observable toxicity, and all hematological and biochemical parameters were found to be within the normal range [48]. Another study assessed the acute and sub-acute toxicity of hydroethanolic leaf extract of *C. polycephalum* in rats for 24 h and 28 days. It showed that treatment with 1 g/kg, 2 g/kg, and 5 g/kg of extract revealed no lethality in the animals following acute toxicity testing. No significant differences were noted in any hematological parameters or in most biochemical parameters, compared to controls, suggesting that long-term administration is generally safe [49]. No mortality or abnormal behavior was observed in rats receiving an aqueous extract of *C. phlomidis* leaves orally at doses of 200, 400 and 800 mg/kg/day for 90 days. However, mild-to-moderate changes were observed in liver and kidney biochemical markers, which correlated with histopathological findings after high doses [50].

## 8. Conclusions and Future Prospective

Medicinal plants and their phytochemicals have demonstrated a wide range of pharmacological effects and have made a significant contribution to the prevention and therapy of numerous diseases. Traditional medicine is also a valuable resource for designing new treatments. In particular, herbal remedies appear suitable for preventing and treating prevalent lifestyle diseases.

The present work provides an overview of current knowledge regarding the promising pharmacological activities associated with *C. trichotomum* and its various bioconstituents. Although the scientific data on its activities are not extensive, studies conducted over the past twenty years suggest it may play a role in treating many ailments. Its extracts and those of its isolated components have anti-inflammatory, antioxidant, cytotoxic, antiviral, and anti-hypertensive properties, and they are effective in treating metabolic disorders. Its biological activity appears to be associated with its phenylpropanoid, flavonoid, terpenoid, and steroid content. Furthermore, cyclohexylethanoids, anthraquinones, lignans, and alkaloids have been identified in the extracts and may also play a role in the activity of the raw material.

*C. trichotomum* appears to have high potential as a treatment, which encourages further research in this area and the recognition of this species beyond its aesthetic values. However, the data regarding the raw material are still too scarce to meet contemporary standards set for official medicinal products. To ensure appropriate dosing, effectiveness, and safety, any plant substance used for pharmaceutical, dietary, and cosmetic purposes requires detailed chemical analysis, confirmation of its efficacy in in vitro, in vivo, and clinical studies, and standardization using validated analytical methods.

In the face of these demands, the raw material and its products need to be included in clinical studies to confirm their therapeutic significance and justify their use. The current successful reports related to the efficacy of the bioactive ingredients of *C. trichotomum* and its extracts are fragmentary, and most studies have employed a relatively simple methodology. Additionally, most studies have focused on in vitro experiments whose results lack clinical applicability. In future, to clarify the specific biological mechanism, more advanced strategies should be used; preclinical and clinical trials are needed to study the targets of active compounds of *C. trichotomum* extracts. Also, no standardized method exists for obtaining and preparing products to ensure they exhibit sufficient dosage, high activity, durability, and safety. As such, future research should assess the bioavailability, pharmacokinetics, distribution, and metabolism of products obtained from *C. trichotomum* in the human body.
